# Reversible acral hyperpigmentation correlating with serum IgG4 levels in IgG4-related disease

**DOI:** 10.1016/j.jdcr.2026.08.043

**Published:** 2026-09-03

**Authors:** Ravid Inbar, Jonathan D. Ho, Deon Wolpowitz

**Affiliations:** a New York Medical College School of Medicine, Valhalla, New York; b Division of Dermatology, Department of Medicine, The University of the West Indies, Mona Campus, Kingston, Jamaica W.I; c Department of Pathology, The University of the West Indies, Mona Campus, Kingston, Jamaica W.I; d Dermatology and Skin Care Associates, P.C., Wellesley, Massachusetts

**Keywords:** acquired hyperpigmentation, acral hyperpigmentation, B-cell depletion, cutaneous manifestations, disease activity marker, IgG4-related disease, reversible hyperpigmentation, rituximab, serum biomarkers

## Introduction

IgG4-related disease (IgG4-RD) is a systemic fibroinflammatory condition characterized by tumefactive lesions and elevated serum IgG4 levels. It can involve multiple organs including the pancreas, biliary tract, salivary glands, and genitourinary system.^1^ Primary cutaneous manifestations present as erythematous papules, plaques, nodules, or subcutaneous masses, on the head and neck.^2^ Histopathology reveals dense infiltrates rich in IgG4-positive plasma cells, often with eosinophils and fibrosis, consistent with direct involvement of the skin.^2^ Rare secondary cutaneous manifestations may lack heavy plasma cell infiltration and include psoriasis-like eruptions, hypergammaglobulinemic purpura, and urticarial vasculitis.^3^ We report an 81-year-old man with established IgG4-RD who developed new-onset acral hyperpigmentation in close temporal association with marked elevation of serum IgG4. Following rituximab therapy, the hyperpigmentation resolved. The eruption recurred with subsequent IgG4 elevation and again resolved after repeat rituximab. This reproducible pattern suggests that acral hyperpigmentation may represent a cutaneous correlate of IgG4-related disease activity.

## Case report

An 81-year-old man with a history of IgG4-RD, previously diagnosed with supportive serologic findings and biopsy-proven multiorgan involvement, including sclerosing cholangitis, pancreatitis, prostatitis, and submandibular gland sialadenitis, presented to the dermatology clinic (July 2021) for evaluation of discoloration on the palms and soles present for 1 month (June 2021). Examination revealed dark gray-brown macules and patches on the palms and soles, without scale, induration, or textural change and unaffected by wiping with alcohol pads (Fig 1).

**Fig 1:**
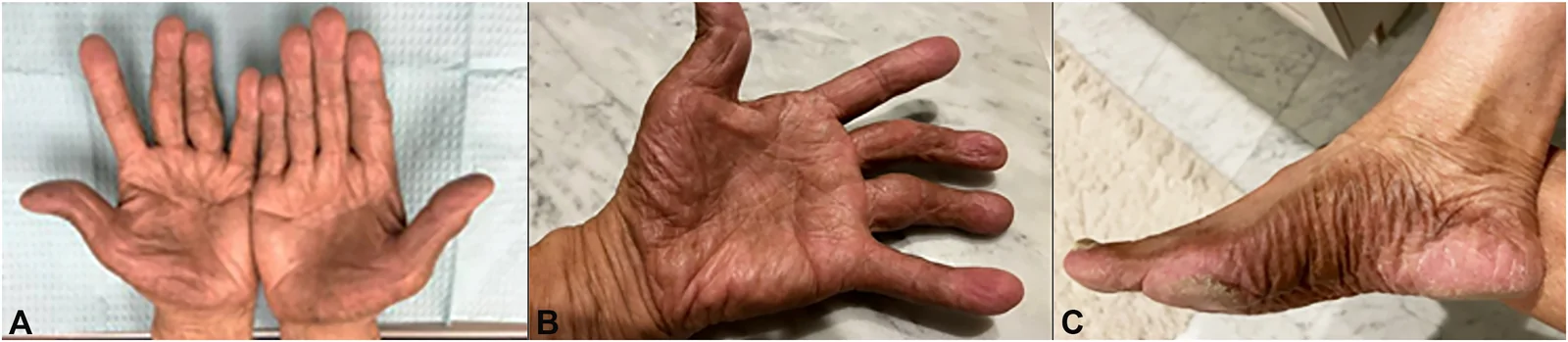
Acral hyperpigmentation. Gray-brown hyperpigmented macules coalescing into patches on the palms and soles at presentation and during recurrence **(A-C)**.

The patient denied well-water consumption, occupational/recreational heavy metal exposure, new medications, or topical exposures. Testing demonstrated normal vitamin B12 levels (359 pg/mL), thyroid-stimulating hormone values from 3.32-5.66 μIU/mL, morning cortisol within normal limits (11.3 μg/dL), and normal white blood cell count (5.88 K/μL), making endocrine causes unlikely. At presentation (July 2021), his serum IgG4 level was markedly elevated at 1215.3 mg/dL (reference range, 3.9-86.4 mg/dL), representing the highest value recorded since a nadir of 176.5 mg/dL in mid-2020 (Fig 2).

**Fig 2:**
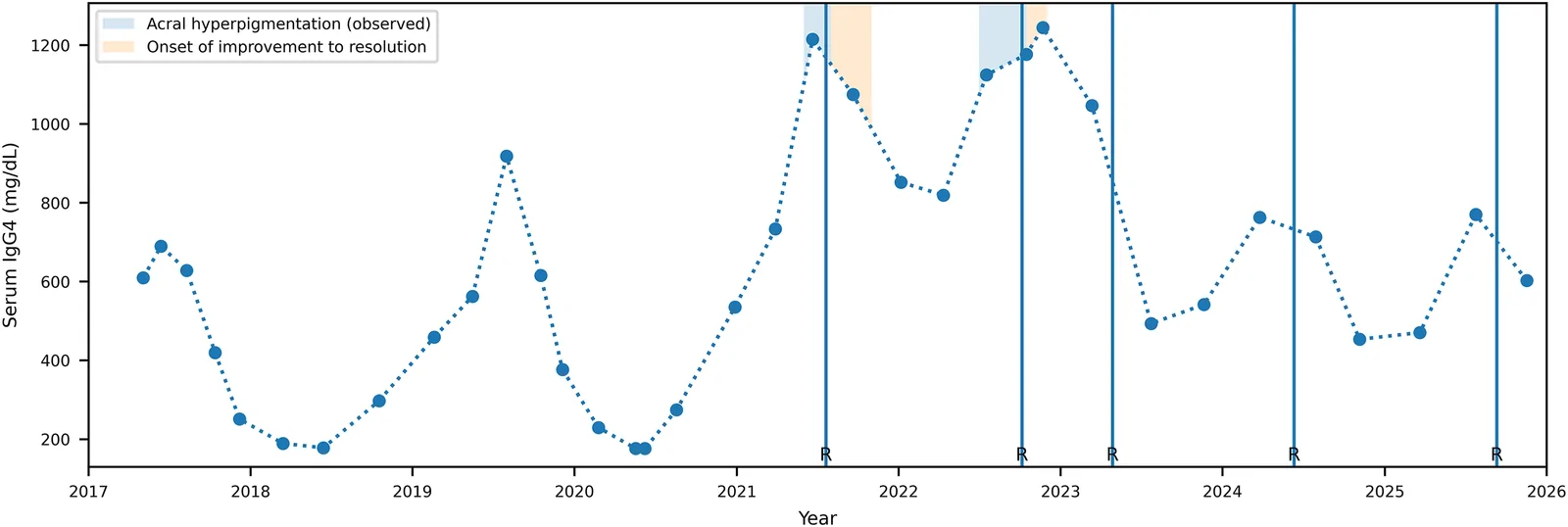
Temporal relationship between serum IgG4 levels and clinical hyperpigmentation. Points represent measured serum IgG4 values; the dotted line represents visual interpolation between measurements. Solid vertical lines indicate rituximab infusion dates. Blue shaded regions denote periods of clinically observed acral hyperpigmentation. Peach shaded regions denote the interval from onset of clinical improvement to resolution of hyperpigmentation.

A punch biopsy of the right palm was performed at presentation (July 2021), revealing basal layer keratinocytic pigmentation, ectatic vessels and a sparse superficial perivascular lymphocytic infiltrate. Fontana–Masson highlighted basal layer pigmentation. Perls stain was negative for hemosiderin. IgG4 immunohistochemistry (monoclonal MRQ-44) revealed extensive dermal staining along with intraepidermal intercellular deposition and perivascular accentuation. True IgG4 deposition is supported by absence of staining in two negative controls: one using an identical staining protocol but without the IgG4 primary antibody on this patient’s sample and one using the complete staining protocol with IgG4 on a biopsy from a patient without IgG4-RD (Fig 3, *A*–*F*).

**Fig 3:**
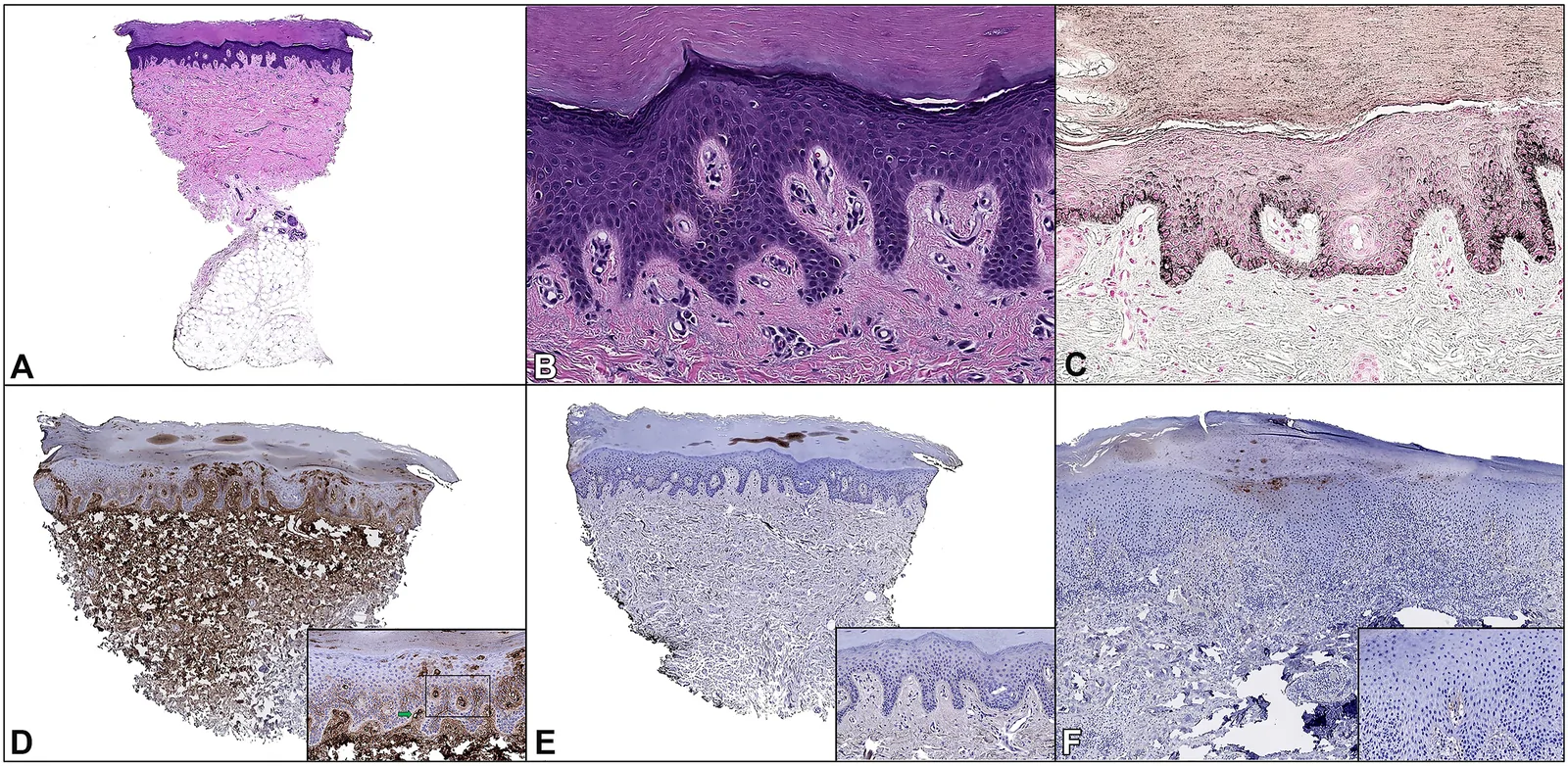
Acral hyperpigmentation histopathology. **A** and **B,** Hematoxylin and eosin staining (2×, 20×) of pigmented area from right palm showed basal layer keratinocytic pigmentation, ectatic blood vessels and a sparse superficial perivascular lymphocytic infiltrate. **C,** Fontana-Masson staining highlights the basal layer keratinocytic pigmentation (20×). **D,** IgG4 immunohistochemistry (4×) highlights extensive background dermal staining (reported in patients with IgG4-related disease) along with (D, inset, 20×) intraepidermal, intercellular deposition (*box*) and accentuation around papillary dermal vessels (*green arrow*). **E,** Note absent staining in both this patient's internal negative control (using an identical staining protocol *without* the IgG4 primary antibody, 4x, inset 20×) and an external negative control (using an identical staining protocol *with* the IgG4 primary antibody, 4x, inset 20x) **(F)** from a patient without IgG4-related disease supporting true deposition of IgG4 in our patient's sample.

He received rituximab 1000 mg intravenously 10 days following biopsy and reported onset of lightening of acral hyperpigmentation within days of the infusion, with marked improvement at 8 weeks (September 2021) and complete resolution of the discoloration by November 2021 (Fig 4, *A*). Subsequently, serum IgG4 levels declined from peak levels but remained elevated at 819.4 mg/dL on April 12, 2022 without recurrence of acral hyperpigmentation (Fig 2).

**Fig 4:**
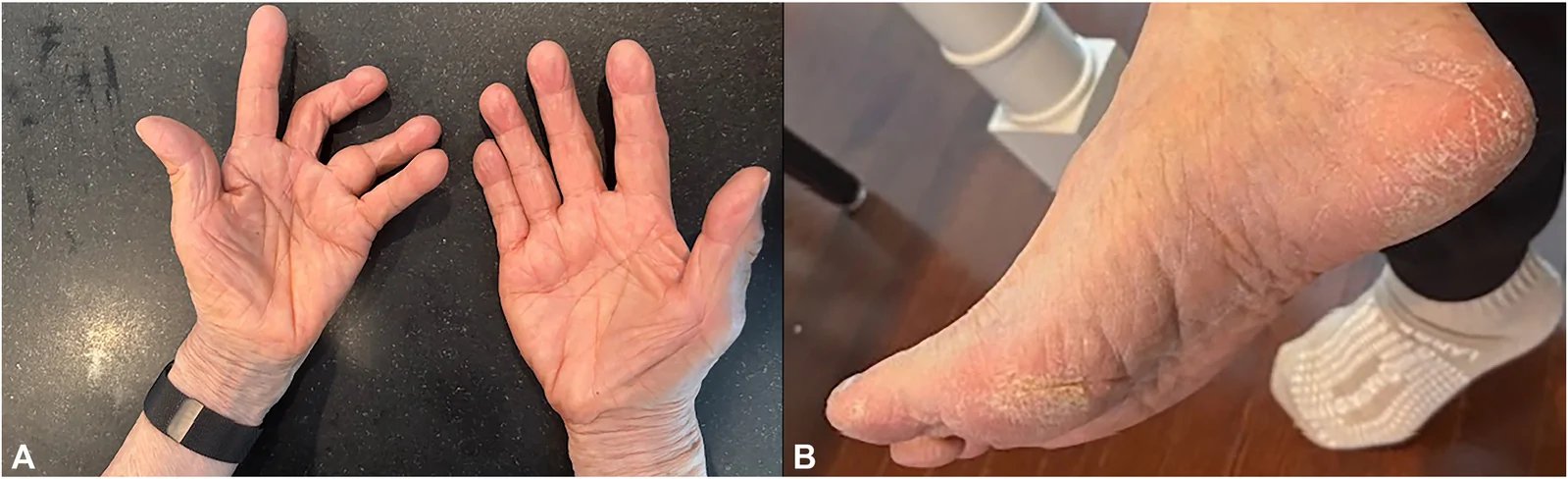
Resolution of acral pigmentation after initial **(A)** and repeat **(B)** rituximab infusions.

In July 2022, similar palmoplantar hyperpigmentation recurred, and the serum IgG4 level had again risen to 1124.6 mg/dL on July 18, 2022. The pigmentation persisted until the patient received repeat rituximab 1000 mg intravenously in October 2022. Again, he reported visible improvement of acral hyperpigmentation within days of this infusion and experienced near complete resolution of acral hyperpigmentation by December 2022 (Fig 4, *B*). While serum IgG4 value was not measured at that time, the acral hyperpigmentation was still resolved when next tested in March 2023 (1046.9 mg/dL). He continued to demonstrate fluctuations in serum IgG4 levels and received additional rituximab infusions (April 2023, June 2024, and September 2025), with corresponding reductions in IgG4 levels following treatment (Fig 2). Importantly, over this period, acral hyperpigmentation did not recur, and subsequent serum IgG4 levels remained below the flare-associated peak values (approximately ≥1100 mg/dL).

## Discussion

We describe a patient with IgG4-RD exhibiting reversible acral hyperpigmentation, temporally associated with marked elevations in serum IgG4, that resolved reproducibly after rituximab therapy. Acral hyperpigmentation, to our knowledge, has not been reported as a cutaneous manifestation of IgG4-RD. In this case, the absence of an associated dense lymphoplasmacytic infiltrate raises the possibility that acral hyperpigmentation is a novel secondary cutaneous manifestation of IgG4-RD. The differential diagnosis of acral hyperpigmentation includes endocrine disorders including Addison disease, iron overload states, medication-induced dyspigmentation, heavy metal exposure, pigment incontinence or postinflammatory hyperpigmentation, and porphyrias. These entities were excluded given normal cortisol and thyroid function testing, negative iron staining, absence in the skin biopsy of lichenoid interface dermatitis changes or papillary dermal melanophages, focal acral distribution of the hyperpigmentation sparing sun-exposed areas, and absence of associated scarring, hypertrichosis, and blisters.

The mechanism underlying this hyperpigmentation is uncertain. Like other secondary manifestations, IgG4 is present, and in this biopsy, is deposited intercellularly between keratinocytes and in the dermis with perivascular accentuation and, like previously reported, as more diffuse and intense background staining likely due to suffusing serum IgG4 deposition.^4^ Whether IgG4 deposition itself drives hyperpigmentation is debatable. The relatively rapid resolution of acral pigmentation compared to the more gradual decline in serum IgG4 levels supports an indirect mechanism over direct mechanistic involvement of immunoglobulin deposition. In this regard, improved cutaneous hyperpigmentation in POEMS (polyneuropathy, organomegaly, endocrinopathy, M-protein, and skin changes) syndrome has been reported in association with declining serum vascular endothelial growth factor levels.^5^ Similarly, in IgG4-related disease, exceeding threshold levels of a Th2/Treg-skewed cytokine milieu (including interleukin (IL)-4, IL-10, IL-13, and transforming growth factor beta^1^^,^^6^ may create a locally altered cutaneous microenvironment that indirectly enhances melanocyte activity and melanosome production, resulting in epidermal hyperpigmentation despite the presence of only sparse perivascular lymphocytic inflammation.

Rituximab is an established therapy for IgG4-RD, inducing rapid peripheral B-cell depletion (over 1-3 days, lasting 2-3 months), resulting in clinical improvement.^7^ In this case, the rapid, reproducible improvement of acral hyperpigmentation following rituximab suggest that this cutaneous finding may serve as a clinical correlate of systemic IgG4-RD activity. Limitations of this case report include derivation from a single patient and lack of a clear pathomechanism. Nevertheless, the reproducible temporal relationship over multiple disease flares supports the proposed association. Further, recognition of this association may prompt consideration of IgG4-RD in patients with otherwise unexplained acral hyperpigmentation.

## Conflicts of interest

None disclosed.
